# Supplementary material for: Analysis of PPARγ Signaling Activity in Psoriasis
Source: Int J Mol Sci. 2021 Aug 10;22(16):8603. doi: 10.3390/ijms22168603 (PMC8395241; doi:10.3390/ijms22168603)
Supplement: Supplementary file 1 [file ijms-22-08603-s001.zip › Supplemental materials_Analysis of PPARg signaling activity in psoriasis/Pathway models/Models images and html files/PPARG inhibits phenotypes assotiated with psoriasis/104540.html]

hemorrhage


# Disease hemorrhage

|  |  |
| --- | --- |
| URN | urn:agi-meshdis:Hemorrhage |
| Total Entities | 61 |
| Connectivity | 7944 |
| Name | hemorrhage |

---

|  |  |
| --- | --- |
| ChildConcepts | experimental hemorrhage |
|  | hemorrhagic stroke |
|  | posterior fossa hemorrhage |
|  | intracerebral hemorrhage |
|  | traumatic brain stem hemorrhage |
|  | hematoma |
|  | hematomyelia |
|  | subarachnoid hemorrhage |
|  | adrenal hemorrhage |
|  | obstetric hemorrhage |
|  | application site bleeding |
|  | urinary tract hemorrhage |
|  | breast hemorrhage |
|  | conjunctival hemorrhage |
|  | subendocardial hemorrhage |
|  | exsanguination |
|  | fetal hemorrhage |
|  | hemorrhagic arthritis |
|  | hemorrhagic conjunctivitis |
|  | hemorrhagic fever |
|  | hemorrhagic hypotension |
|  | hemorrhoid hemorrhage |
|  | digestive hemorrhage |
|  | injection site hemorrhage |
|  | lip hemorrhage |
|  | respiratory tract hemorrhage |
|  | meningeal hemorrhage |
|  | genital hemorrhage |
|  | hemorrhagic diathesis |
|  | mucosal hemorrhage |
|  | muscle hemorrhage |
|  | neonatal hemorrhage |
|  | pharyngeal hemorrhage |
|  | retroperitoneal hemorrhage |
|  | cutaneous bleeding |
|  | intraocular hemorrhage |
|  | tumor hemorrhage |
|  | wound hemorrhage |
|  | hemarthrosis |
|  | epistaxis |
|  | gingival hemorrhage |
|  | hematemesis |
|  | hemoperitoneum |
|  | oral hemorrhage |
|  | hemorrhagic shock |
|  | Shwartzman phenomenon |
|  | intraoperative blood loss |
|  | postoperative hemorrhage |
|  | retrobulbar hemorrhage |
|  | traumatic intracranial hemorrhage |
|  | hypertensive intracranial hemorrhage |
|  | hemorrhagic infarction |
|  | subconjunctival hemorrhage |
|  | internal hemorrhage |
|  | hemopericardium |
|  | abdominal hemorrhage |
|  | hemorrhagic complication |
|  | hemophilic pseudotumor |
|  | hemorrhagic transformation |
|  | diffuse alveolar hemorrhage |
|  | variceal hemorrhage |

---

|  |  |
| --- | --- |
| Pathway | PPARG inhibits phenotypes assotiated with psoriasis |
|  | 1\_2\_Feedback relations between coagulation and fibrinolysis |
|  | 1\_1\_Dysfunction of coagulation cascade in hemophilia |

---

|  |  |
| --- | --- |
| MedScan ID | 9003084 |

---

|  |  |
| --- | --- |
| Alias | Hemorrhage Terms |
|  | hemorrhagic activity |
|  | hemorrhagias |
|  | hemorrhage models |
|  | Hemorrhage |
|  | Blood Loss |
|  | loss of blood |
|  | hemorrhage model |
|  | Bleeding |
|  | losses of blood |
|  | rebleedings |
|  | hemorrhagic activities |
|  | Extravasation of Blood |
|  | Hemorrhages |
|  | Blood Losses |
|  | re-bleeding |
|  | hemorrhagia |
|  | rebleeding |
|  | hemorrhagic events |
|  | Hemorrhage Term |
|  | bleedings |
|  | spontaneous hemorrhage |
|  | hemorrhagic event |
|  | haemorrhagic event |
|  | spontaneous haemorrhage |
|  | haemorrhagic events |
|  | Haemorrhages |
|  | Haemorrhage |
|  | haemorrhage models |
|  | haemorrhage model |
|  | haemorrhagic activities |
|  | haemorrhagic activity |

---

|  |  |
| --- | --- |
| MeSH ID | D006470 |
|  | M0010152 |

---

|  |  |
| --- | --- |
| MeSH Heading | Hemorrhage |

---
